# Reduced Prostasin (CAP1/PRSS8) Activity Eliminates HAI-1 and HAI-2 Deficiency–Associated Developmental Defects by Preventing Matriptase Activation

**DOI:** 10.1371/journal.pgen.1002937

**Published:** 2012-08-30

**Authors:** Roman Szabo, Katiuchia Uzzun Sales, Peter Kosa, Natalia A. Shylo, Sine Godiksen, Karina K. Hansen, Stine Friis, J. Silvio Gutkind, Lotte K. Vogel, Edith Hummler, Eric Camerer, Thomas H. Bugge

**Affiliations:** 1 Oral and Pharyngeal Cancer Branch, National Institute of Dental and Craniofacial Research, National Institutes of Health, Bethesda, Maryland, United States of America; 2 Department of Cellular and Molecular Medicine, Faculty of Health Science, University of Copenhagen, Copenhagen, Denmark; 3 Department of Biology, Faculty of Science, University of Copenhagen, Copenhagen, Denmark; 4 Pharmacology and Toxicology Department, University de Lausanne, Lausanne, Switzerland; 5 INSERM U970, Paris Cardiovascular Research Centre, Paris, France; 6 Université Paris-Descartes, Paris, France; SA Pathology, Australia

## Abstract

Loss of either hepatocyte growth factor activator inhibitor (HAI)-1 or -2 is associated with embryonic lethality in mice, which can be rescued by the simultaneous inactivation of the membrane-anchored serine protease, matriptase, thereby demonstrating that a matriptase-dependent proteolytic pathway is a critical developmental target for both protease inhibitors. Here, we performed a genetic epistasis analysis to identify additional components of this pathway by generating mice with combined deficiency in either HAI-1 or HAI-2, along with genes encoding developmentally co-expressed candidate matriptase targets, and screening for the rescue of embryonic development. Hypomorphic mutations in *Prss8*, encoding the GPI-anchored serine protease, prostasin (CAP1, PRSS8), restored placentation and normal development of HAI-1–deficient embryos and prevented early embryonic lethality, mid-gestation lethality due to placental labyrinth failure, and neural tube defects in HAI-2–deficient embryos. Inactivation of genes encoding c-Met, protease-activated receptor-2 (PAR-2), or the epithelial sodium channel (ENaC) alpha subunit all failed to rescue embryonic lethality, suggesting that deregulated matriptase-prostasin activity causes developmental failure independent of aberrant c-Met and PAR-2 signaling or impaired epithelial sodium transport. Furthermore, phenotypic analysis of PAR-1 and matriptase double-deficient embryos suggests that the protease may not be critical for focal proteolytic activation of PAR-2 during neural tube closure. Paradoxically, although matriptase auto-activates and is a well-established upstream epidermal activator of prostasin, biochemical analysis of matriptase- and prostasin-deficient placental tissues revealed a requirement of prostasin for conversion of the matriptase zymogen to active matriptase, whereas prostasin zymogen activation was matriptase-independent.

## Author Summary

Vertebrate embryogenesis is dependent upon a series of precisely coordinated cell proliferation, migration, and differentiation events. Recently, the execution of these events was shown to be guided in part by extracellular cues provided by focal pericellular proteolysis by a newly identified family of membrane-anchored serine proteases. We now show that two of these membrane-anchored serine proteases, prostasin and matriptase, constitute a single proteolytic signaling cascade that is active at multiple stages of development. Furthermore, we show that failure to precisely regulate the enzymatic activity of both prostasin and matriptase by two developmentally co-expressed transmembrane serine protease inhibitors, hepatocyte growth factor activator inhibitor-1 and -2, causes an array of developmental defects, including clefting of the embryonic ectoderm, lack of placental labyrinth formation, and inability to close the neural tube. Our study also provides evidence that the failure to regulate the prostasin–matriptase cascade may derail morphogenesis independent of the activation of known protease-regulated developmental signaling pathways. Because hepatocyte growth factor activator inhibitor–deficiency in humans is known to cause an assortment of common and rare developmental abnormalities, the aberrant activity of the prostasin–matriptase cascade identified in our study may contribute importantly to genetic as well as sporadic birth defects in humans.

## Introduction

Studies conducted within the past two decades have uncovered a large family of membrane-anchored serine proteases that regulates vertebrate development, tissue homeostasis, and tissue repair by providing focal proteolysis essential for cytokine and growth factor maturation, extracellular matrix remodeling, signaling receptor activation, receptor shedding, regulation of ion channel activity, and more (reviewed in [1], [2], [3]). Individual members of this family regulate both vertebrate development and postnatal tissue homeostasis, including auditory and vestibular system development [4], [5], [6], differentiation of stratified epithelia [7], [8], loss of epithelial tight junction function [9], [10], failure to activate digestive enzymes [11], thyroid hormone availability [4], sodium and water homeostasis [12], [13], [14], iron homeostasis [15], [16], and fertility [17], [18]. Likewise, mounting evidence suggests that excessive or spatially dysregulated membrane-anchored serine protease activity contributes to several human disorders, including congenital malformations [19], epithelial dysfunction [20], [21], [22], and cancer [3].

Matriptase is a modular type II transmembrane serine protease, encoded by the *ST14* gene, that has pleiotropic functions in epithelial development and postnatal homeostasis, at least in part through its capacity to regulate epithelial tight junction formation in simple and stratified epithelia [2], [3]. In the human and mouse epidermis, matriptase appears to function as part of a proteolytic cascade in which it acts upstream of the GPI-anchored serine protease prostasin (CAP1/PRSS8), most likely by directly activating the prostasin zymogen [23], [24], [25], [26]. Several additional candidate proteolytic substrates have been identified for matriptase in cell-based and biochemical assays, including growth factor precursors [27], [28], [29], [30], protease-activated signaling receptors [31], [32], [33], ion channels [34], [35], and other protease zymogens besides pro-prostasin [29], [36], [37]. However, the extent to which cleavage of these substrates is critical to matriptase-dependent epithelial development and maintenance of epithelial homeostasis needs to be established.

Although matriptase is not required for term development in humans and most mouse strains ([24], [38], and Szabo et al., unpublished data), the membrane-anchored serine protease nevertheless is expressed in many burgeoning embryonic as well as extraembryonic epithelia [39], [40], [41], [42]. Furthermore, we have previously shown that matriptase must be tightly regulated at the post-translational level, for successful execution of several developmental processes. Thus, loss of either of the two Kunitz-type transmembrane serine protease inhibitors, hepatocyte growth factor activator inhibitor (HAI)-1 or -2 or combined haploinsufficiency for both inhibitors, is associated with uniform embryonic lethality in mice [40], [43]. Loss of HAI-1 or combined haploinsufficiency for HAI-1 and HAI-2 causes mid-gestation embryonic lethality due to failure to develop the placental labyrinth. Loss of HAI-2, in turn, is associated with three distinct phenotypes: a) Early embryonic lethality, b) mid-gestation lethality due to placental labyrinth failure, and c) neural tube defects resulting in exencephaly, spina bifida, and curly tail. All developmental defects in HAI-1- and HAI-2-deficient embryos, however, are rescued in whole or in part by simultaneous matriptase-deficiency, thus demonstrating that a matriptase-dependent proteolytic pathway is a critical morphogenic target for both protease inhibitors ([43], [44], this study).

In this study, we exploited the observation that HAI-1- and HAI-2-deficient mice display matriptase-dependent embryonic lethality with complete penetrance to perform a comprehensive genetic epistasis analysis aimed at identifying additional components of the matriptase proteolytic pathway. Specifically, we generated mice with simultaneous ablation of either the *Spint1* gene (encoding HAI-1) or the *Spint2* gene (encoding HAI-2) along with genes encoding candidate matriptase targets that are co-expressed with the protease during development. We then screened for the rescue of embryonic lethality or restoration of HAI-1 and HAI-2-dependent morphogenic processes in these double-deficient mice. This analysis identified prostasin as critical to all matriptase-induced embryonic defects in both HAI-1- and HAI-2-deficient mice. Paradoxically, however, although matriptase autoactivates efficiently and prostasin is incapable of undergoing autoactivation, we found that prostasin acts upstream of matriptase in the developing embryo and is required for conversion of the matriptase zymogen to active matriptase. Finally, we explored the contribution of this newly identified prostasin-matriptase pathway to protease-activated receptor (PAR)-dependent signaling during neural tube formation [45] and now provide evidence that the pathway may be separate from the proteolytic machinery that mediates focal activation of PAR-2 during neural tube closure.

## Results

### Developmental defects in HAI-2–deficient mice tightly correlate with matriptase expression levels

**Figure 1:**
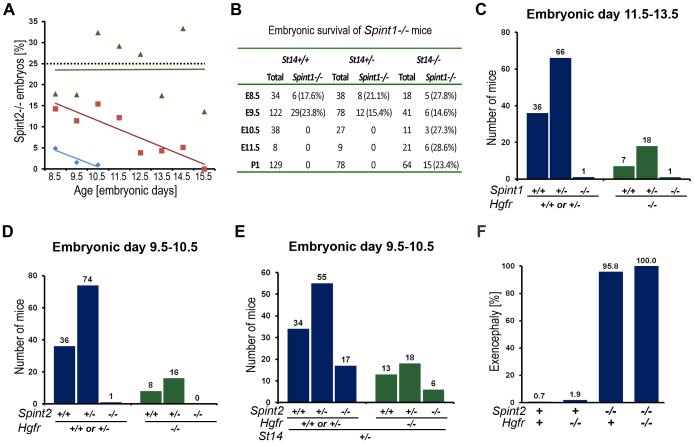
Effect of *St14* gene dosage and c-Met activity on embryonic development in HAI-1– and HAI-2–deficient mice. (A) Matriptase haploinsufficiency partially restores early embryonic development of HAI-2 deficient mice. Relative frequency of *Spint2^−/−^*;*St14^+/+^* (blue diamonds and trend line), *Spint2^−/−^*;*St14^+/−^* (red squares and trendline), and *Spint2^−/−^*;*St14^−/−^* (green triangles and trendline) embryos in offspring from interbred *Spint2* ^+/−^ *;St14^+/−^* mice at E8.5–E15.5. The expected 25% Mendelian frequency is shown with the dotted trend line. 59–250 embryos were genotyped at each stage. (B) Matriptase haploinsufficiency does not rescue development of HAI-1-deficient mice. Genotype distribution of E8.5–E11.5 embryos and newborn (P1) offspring from interbred *Spint1* ^+/−^ *;St14^+/−^* mice. No living *St14^+/+^*;*Spint1* ^−/−^ or *St14^+/−^*;*Spint1* ^−/−^ embryos are observed after E9.5. (C) Distribution of *Spint1* genotypes in c-Met-expressing (*Hgfr^+/+^* or *Hgfr^+/−^*, blue bars) and c-Met-deficient (*Hgfr^−/−^*, green bars) embryos from interbred *Spint1* ^+/−^ *;Hgfr^+/−^* mice at E11.5–13.5. Loss of c-Met activity does not improve embryonic survival of HAI-1-deficient mice. (D and E) Distribution of *Spint2* genotypes in c-Met-expressing (*Hgfr^+/+^* or *Hgfr^+/−^*, blue bars) and c-Met-deficient (*Hgfr^−/−^*, green bars) embryos from *Spint2* ^+/−^ *;Hgfr^+/−^*×*Spint2* ^+/−^ *;Hgfr^+/−^* (D) or *Spint2* ^+/−^ *;Hgfr^+/−^*×*Spint2* ^+/−^ *;Hgfr^+/−^;St14^+/−^* (E) breeding pairs at E9.5–10.5. Only *St14^+/−^* embryos are shown in (E). Loss of c-Met does not improve survival of HAI-2-deficient embryos. (F) Frequency of exencephaly observed in 153 control (*Spint2^+^;Hgfr^+^*), 53 c-Met- (*Spint2^+^;Hgfr^−/−^*), 24 HAI-2- (*Spint2^−/−^,Hgfr^+^*), and 6 c-Met and HAI-2 double- (*Spint2^−/−^;Hgfr^−/−^*) deficient embryos at E9.5. Loss of c-Met activity fails to correct neural tube defects in HAI-2-deficient mice.

HAI-2-deficient (*Spint2^−/−^*) mice were originally reported to display embryonic lethality prior to embryonic day 8 (E8.0), presenting with severe clefting of the embryonic ectoderm at E7.5 and a failure to progress to the headfold stage [44]. We previously reported, however, that approximately 50% of HAI-2-deficient mice complete early development but die at midgestation due to defective placental branching morphogenesis [43]. However, the genotyping strategy used in the latter study aimed at exploring the contribution of matriptase to this embryonic demise and only allowed for the discrimination of HAI-2-deficient mice on matriptase-sufficient (wildtype, *Spint2^−/−^;St14^+/+^*, or haploinsufficient, *Spint2^−/−^;St14^+/−^*) backgrounds from a matriptase-deficient (*Spint2^−/−^;St14^−/−^*) background. Therefore, to test the possibility that early embryonic development of HAI-2-deficient mice is *St14* gene dosage-dependent, we first analyzed the offspring of interbred *Spint2^+/−^*;*St14^+/−^* mice at various developmental stages. This analysis revealed that the various developmental phenotypes seen in HAI-2-deficient mice, indeed, were strongly dependent on *St14* gene dosage (Figure 1A). Thus, HAI-2-deficient embryos carrying two wildtype matriptase alleles (*St14^+/+^*), displayed early lethality, as evidenced by only five percent of *Spint2^−/−^;St14^+/+^* embryos developing beyond E9.0 and none past E10.5 (Figure 1A, blue diamonds). Inactivation of one matriptase allele (*Spint2^−/−^;St14^+/−^*), however, was sufficient to partially rescue this early embryonic lethality of HAI-2-deficient mice (Figure 1A, red squares). As reported previously [43], inactivation of both alleles of matriptase (*Spint2^−/−^;St14^−/−^*) completely restored embryonic survival and placental development and also reduced the occurrence of neural tube defects associated with the loss of HAI-2 (Figure 1A, green triangles and Table 1). Taken together, these findings show that loss of HAI-2 may lead to three distinct developmental phenotypes, dependent on the overall expression level of matriptase (Table 1): (i) early embryonic lethality occurring largely prior to E8.5, which can be partially rescued by matriptase haploinsufficiency (*Spint2^−/−^;St14^+/−^*) and completely by matriptase deficiency (*Spint2^−/−^;St14^−/−^*); (ii) placental defects resulting in mid-gestation lethality, which are observed in *Spint2^−/−^;St14^+/−^* embryos after E9.5, but are absent in *Spint2^−/−^;St14^−/−^* embryos, and (iii) neural tube defects observed at or after E8.5 in most *Spint2^−/−^;St14^+/−^* embryos, and partially rescued in *Spint2^−/−^;St14^−/−^* embryos and term offspring.

**Table 1:** Developmental defects observed in *Spint1*- and *Spint-2*-deficient mice as function of *St14* expression.

Genotype	Phenotype	Penetrance		
		*St14^+/+^*	*St14^+/−^*	*St14^−/−^*
***Spint1^−/−^***	Lack of placental labyrinth, embryonic lethality at E10.5	100%	100% (no rescue)	0% (complete rescue)
***Spint2^−/−^***	Early embryonic lethality at E9.5 or earlier	100%	45% (partial rescue)	0% (complete rescue)
	Incomplete differentiation of placental labyrinth, embryonic lethality at E10.5–E14.5	N/A	100% (no rescue)	0% (complete rescue)
	Neural tube defects	N/A		
	Exencephaly		95–100%	18% (partial rescue; P<0.0001)
	Spina bifida		11%	13% (no rescue)
	Curly tail		89%	62% (partial rescue?; not significant)

We next performed a similar analysis of the effect of *St14* gene dosage on the developmental defects and embryonic lethality associated with HAI-1-deficiency by analyzing the offspring from interbred *Spint1^+/−^*/*St14^+/−^* mice (Figure 1B). As shown previously [40], a complete rescue of both the placental defects and embryonic lethality was observed in HAI-1-deficient mice expressing no matriptase (*Spint1^−/−^;St14^−/−^*). However, comparison of HAI-1-deficient mice carrying one (*Spint1^−/−^;St14^+/−^*) or two (*Spint1^−/−^;St14^+/+^*) wildtype *St14* alleles revealed identical defects in placental labyrinth formation and mid-gestation embryonic lethality occurring with complete penetrance (Figure 1B, Table 1, and data not shown).

### Activation of hepatocyte growth factor (HGF) does not contribute to placental defects in HAI-1–deficient embryos or early embryonic lethality and neural tube defects in HAI-2–deficient mice

Matriptase is an efficient activator of proHGF [29], [30] and dysregulated matriptase activity recently was shown to promote squamous cell carcinoma through activation of HGF-dependent c-Met signaling [46]. Furthermore, both proHGF and its cognate receptor c-Met are expressed during embryogenesis in both the placenta and the embryo [47], [48]. To investigate the involvement of aberrant proHGF activation and c-Met signaling in the etiology of the defects observed in HAI-1- and HAI-2-deficient embryos, we took advantage of the fact that c-Met is only required for embryonic development beyond E13.5 [47], [48]. This enabled the study of key HAI-1- and HAI-2-dependent morphogenic processes in mice homozygous for a null mutation in *Hgfr* (*Hgfr^−/−^*), encoding c-Met. Analysis of embryos from interbred *Spint1^+/−^;Hgfr^+/−^* mice at E11.5–E13.5 revealed only one surviving *Spint1^−/−^;Hgfr^−/−^* embryo, indicating that the loss of c-Met activity does not restore placental development or embryonic survival of HAI-1-deficient mice (Figure 1C, P<0.04, Chi-square test, and data not shown). Likewise, no *Spint2^−/−^;Hgfr^−/−^* embryos were detected beyond E9.5 (Figure 1D, P<0.02, Chi-square test), indicating that the inactivation of c-Met signaling does not prevent matriptase-induced early embryonic lethality in HAI-2-deficient mice. Interbreeding *Spint2^+/−^;Hgfr^+/−^;St14^+/−^* mice allowed for the analysis of the impact of c-Met deficiency on the formation of neural tube defects in HAI-2-deficient mice by preventing early embryonic lethality (Figure 1E). However, all of the *Spint2^−/−^;Hgfr^−/−^;St14^+/−^* embryos isolated at E9.5 from these crosses presented with exencephaly (Figure 1F), suggesting that c-Met signaling is not critically involved in the neural tube defects caused by the absence of HAI-2. All *Spint2^−/−^;Hgfr^−/−^;St14^+/−^* embryos displayed synthetic lethality after E9.5, which precluded the direct analysis of the impact of c-Met loss on the defects in placental differentiation caused by HAI-2 deficiency (data not shown). Taken together, these findings suggest that aberrant HGF-c-Met signaling does not contribute to the matriptase-dependent defects in placentation in HAI-1-deficient embryos, or early lethality and neural tube closure of HAI-2-deficient embryos.

### Reduced prostasin enzymatic activity prevents developmental defects in both HAI-1– and HAI-2–deficient mice

**Figure 2:**
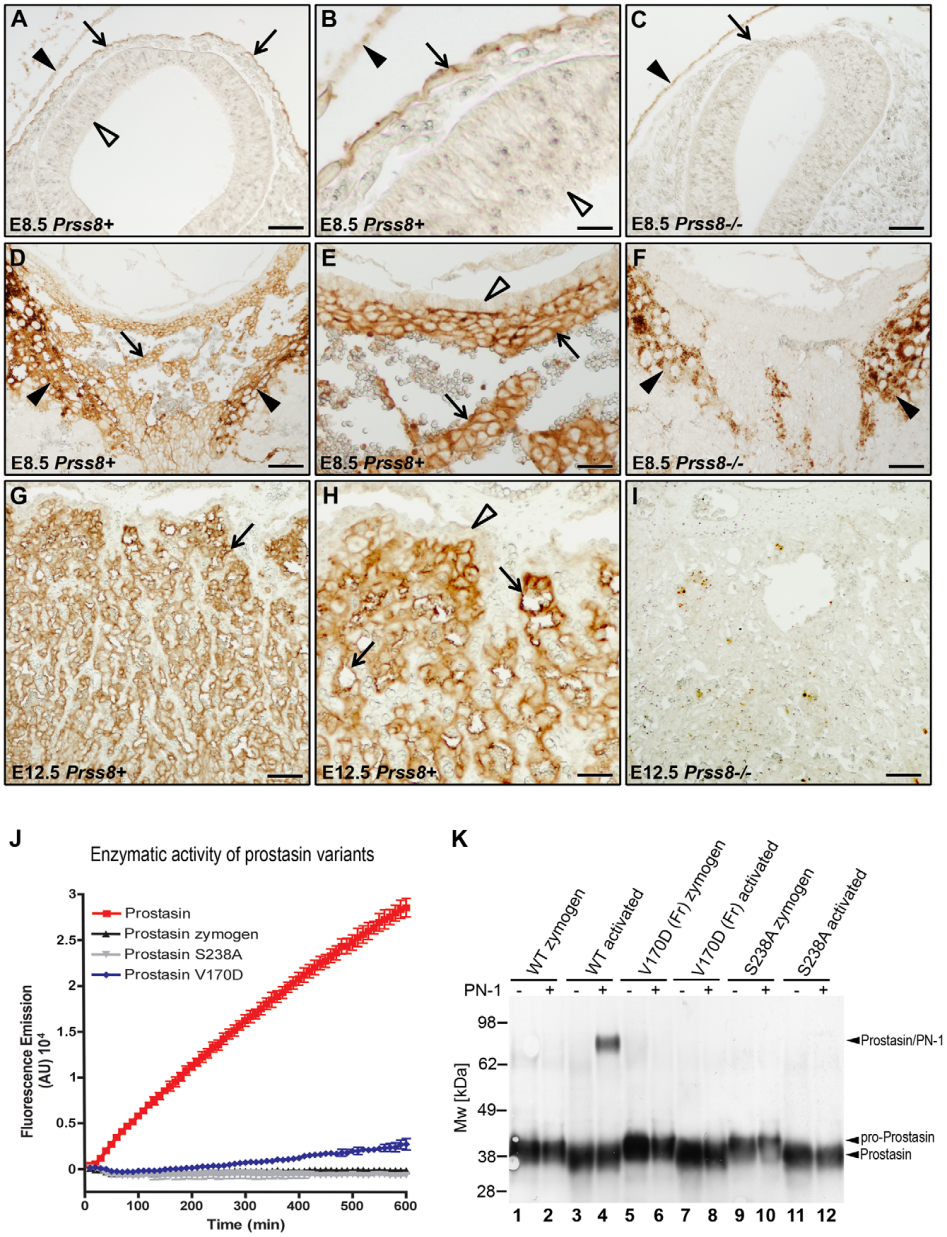
Prostasin expression in embryonic and extraembryonic tissues. (A–C) Immunohistochemical detection of prostasin at E8.5 in epithelial cells of surface ectoderm (examples with arrows in A and B) overlying the cranial neural tube region. Specificity of staining is shown by the absence of staining of *Prss8^−/−^* surface ectoderm (arrow in C). Filled arrowhead shows non-specific staining of yolk sac. No expression was observed in the neuroepithelium (A and B, open arrowheads). (D–F) Immunohistochemical detection of prostasin in the chorionic ectoderm (examples with arrows) of mouse placenta at E8.5. Specificity of staining is shown by the absence of staining of *Prss8^−/−^* chorionic ectoderm (F). Filled arrowheads in D and F shows non-specific staining of trophoblast giant cells. No expression was detected in the trophoblast stem cell-containing chorionic epithelium (open arrowhead in E). (G–I) Immunohistochemical detection of prostasin in the placental labyrinth (examples with arrows in G and H) of mouse placenta at E12.5. Specificity of staining is shown by the absence of staining of the *Prss8^−/−^* labyrinth (I). No expression was detected in the trophoblast stem cell-containing chorionic epithelium (open arrowhead in H). Scale bars: A, C, D, F, G, and I, 100 µm; B, E, and H, 25 µm. (J) Enzymatic activity of wildtype (red), V170D (blue), S238A (grey), and zymogen (black) forms of prostasin. Prostasin variants were incubated with 50 µM pERTKR-AMC fluorogenic peptide at 37°C. V170D prostasin exhibited about 6% of the amidolytic activity of wildtype prostasin. No activity of catalytically inactive prostasin or prostasin zymogen was detected. (K) Western blot detection of SDS-stable complexes between prostasin and protein nexin-1 (PN-1). Wildtype zymogen (lanes 1 and 2), activated wildtype (lanes 3 and 4), V170D (frizzy) zymogen (lanes 5 and 6), activated V170D (lanes 7 and 8), S238A zymogen (lanes 9 and 10), and activated S238A (lanes 11 and 12) prostasin variants were incubated with (lanes 2, 4, 6, 8, 10, and 12) or without (lanes 1, 3, 5, 7, 9, and 11) 250 ng of recombinant human PN-1. Wildtype, but not V170D or S238A variants of prostasin formed SDS-stable complexes with PN-1. Positions of pro-prostasin, activated prostasin (migrating slightly faster than the zymogen due to removal of the 12 aa propeptide that is not detected after 4–12% SDS/PAGE with anti-prostasin antibody), and prostasin/PN-1 complexes are indicated. Positions of molecular weight markers (kDa) are shown on left.

**Figure 3:**
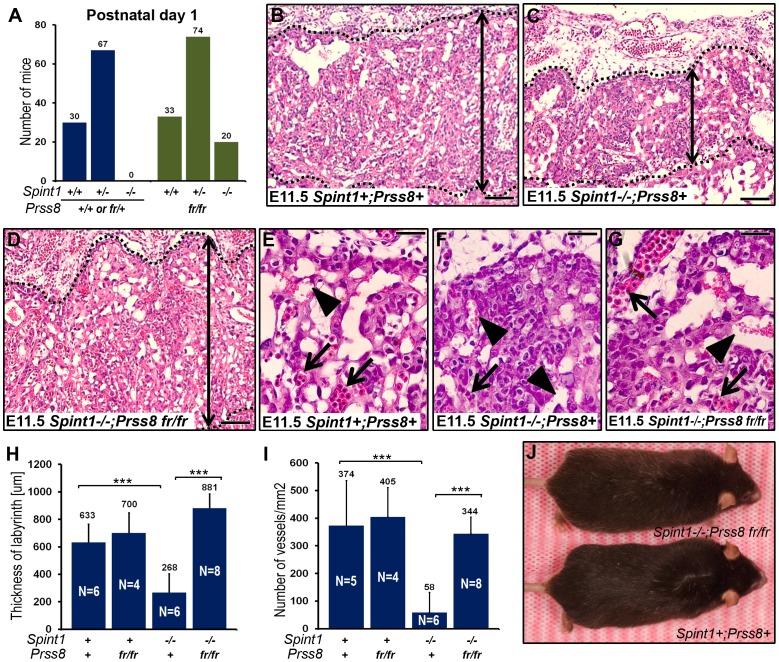
Reduced prostasin activity restores placental development and embryonic survival of HAI-1–deficient mice. (A) Distribution of genotypes of born offspring of intercrossed *Spint1* ^+/−^ *;Prss8^fr/−^* mice. No *Spint1^−/−^* mice expressing one or two wildtype prostasin alleles (*Prss8^+/+^* or *Prss8^+/fr^*, blue bars) were identified, while *Spint1^−/−^* embryos carrying two mutant prostasin alleles (*Prss8^fr/fr^*, green bars) were found in near-expected frequency. (B–G) Representative low (B–D) and high (E–G) magnification images showing the histological appearance of H&E-stained placental tissues of (*Spint1^+^;Prss8^+^*) (B and E), (*Spint1^−/−^;Prss8^+^*) (C and F), and (*Spint1^−/−^;Prss8^fr/fr^*) (D and G) embryos at E11.5. The thickness of the placental labyrinth (two-sided arrows between the dotted lines in B–D), as well as the number of fetal vessels (E–G, arrows) and lacunae filled with maternal blood (E–G, arrowheads) within the labyrinth is markedly reduced in prostasin-sufficient (C and F), but not in prostasin-deficient (D and G) *Spint1^−/−^* embryos, when compared to the controls (B and E). (H, I) Quantification of the maximum thickness of the labyrinth layer (H) and the number of fetal vessels in the placental labyrinth (I) of *Spint1^+^;Prss8^+^*, *Spint1^+^;Prss8^fr/fr^*, *Spint1^−/−^;Psrr8^+^*, and *Spint1^−/−^;Psrr8^fr/fr^* embryos at E11.5. The thickness of the labyrinth and fetal vessel density were strongly diminished in HAI-1-deficient mice but completely restored in HAI-1-deficient mice with low prostasin activity. (J) Outward appearance of one-year-old *Spint1^−/−^;Prss8^fr/fr^* and littermate *Spint1^+^;Prss8^+^* mice. ***, p<0.0001, Student's t-Test, two tailed. Scale bars: B–D, 100 µm; E–G, 25 µm.

The GPI-anchored membrane serine protease, prostasin (CAP1/PRSS8), is a well-validated downstream proteolytic target for matriptase in the epidermis of mice and humans (see Introduction). To explore the possibility that matriptase acts through prostasin to cause the signature defects in embryonic development of HAI-1- and HAI-2-deficient mice, we first performed a detailed immunohistochemical analysis of prostasin expression in the developing embryo by staining histological sections from wildtype (*Prss8^+/+^*) and littermate prostasin-deficient (*Prss8^−/−^*) embryos with prostasin antibodies. Interestingly, prostasin was expressed in both the surface ectoderm, specifically covering the converging neuroepithelium at the time of the neural tube closure (Figure 2A and 2B, compare with 2C), and in the developing placenta, where expression was detected as early as on E8.5 and was present in the placental labyrinth in the entire period of placental differentiation (Figure 2D, 2E, 2G, and 2H, compare with 2F and 2I), thereby displaying co-expression with matriptase, HAI-1 and HAI-2 [40], [41], [42], [43], [45]. We, therefore, next directly determined the contribution of prostasin to the matriptase-dependent developmental defects of HAI-1- and HAI-2-deficient mice. For this purpose, we exploited the fact that the spontaneous mutant mouse strain, *frizzy*, recently was described to be homozygous for a point mutation in the coding region of the *Prss8* gene (*Prss8* ^fr/fr^). This mutation results in a non-conservative V170D amino acid substitution in the prostasin protein [49]. Moreover, this mutant mouse strain completes development, but displays an epidermal phenotype resembling mice carrying a hypomorphic mutation in *St14* [23], suggesting reduced expression or enzymatic activity of V170D prostasin. Western blot and immunohistochemical analysis of tissues from *Prss8^fr/fr^* mice did not reveal an obvious reduction in the level of V170D prostasin expression when compared to wildtype prostasin in *Prss8^+/+^* littermates (data not shown). Therefore, to assess the enzymatic activity of the mutant prostasin, we generated enteropeptidase-activated recombinant V170D prostasin, as well as enteropeptidase-activated wildtype and catalytically inactive (S238A) prostasin variants in HEK293T cells, as described previously [26]. These recombinant proteins were released from the plasma membrane by phosphatidylinositol-specific phospholipase C, activated with enteropeptidase, and their enzymatic activity towards a prostasin-selective fluorogenic peptide substrate (Figure 2J) as well as their ability to form enzymatic activity-dependent covalent complexes with the serpin, protease nexin-1 (PN-1) (Figure 2K), were tested. As expected, wildtype recombinant prostasin exhibited easily detectable hydrolytic activity towards the fluorogenic peptide (Figure 2J, red line) and formed SDS-stable complexes with PN-1 (Figure 2K, compare lanes 3 and 4), while prostasin not activated by enteropeptidase and the catalytically inactive S238A mutant and exhibited no detectable hydrolytic activity (Figure 2J, black and grey lines) or PN-1 binding (Figure 2K and Figure S1A, lanes 2 and 12). V170D prostasin displayed a low residual enzymatic activity that was above the baseline level, as defined by the catalytically inactive S238A variant, and corresponded to about 6% of the activity of wildtype prostasin (Figure 2J, blue line), while complex formation with PN-1 could not be detected (Figure 2K and Figure S1A, compare lanes 7 and 8). Taken together, these data indicated that V170D prostasin, expressed by the *Prss8^fr^* allele, displays greatly reduced enzymatic activity. We, therefore, next interbred *Spint1^+/−^;Prss8^fr/+^* and *Spint1^+/−^;Prss8^fr/fr^* mice and analyzed the distribution of *Spint1* alleles in the newborn offspring from these crosses. Consistent with our previous findings, loss of HAI-1 was not compatible with embryonic survival of mice carrying a wildtype prostasin allele (*Spint1^−/−^;Prss8^fr/+^*) (Figure 3A, blue bars). Interestingly, however, HAI-1-deficient mice carrying two mutant prostasin alleles (*Spint1^−/−^;Prss8^fr/fr^*) developed to term (Figure 3A, green bars), although they were found at a frequency that was slightly lower than the expected Mendelian distribution (20/127, 16% vs. expected 31.75/127, 25%, P<0.05, Chi-square test). Furthermore, morphometric analysis showed that reduced prostasin activity fully restored placental labyrinth formation in HAI-1-deficient embryos, as evidenced by normal histological appearance of the labyrinth (Figure 3B–3G), thickness of the labyrinth layer (Figure 3H) and labyrinth vessel density (Figure 3I) of *Spint1^−/−^;Prss8^fr/fr^* embryos. Furthermore, macroscopic and histological analysis of embryos extracted between E11.5 and E13.5 failed to reveal any obvious developmental abnormalities within either embryonic or extraembryonic tissues of *Spint1^−/−^;Prss8^fr/fr^* mice (data not shown), and these mice were outwardly indistinguishable from their *Prss8^fr/fr^* littermates at weaning and when followed for up to one year (Figure 3J). Taken together, these data show that the matriptase-mediated developmental defects in HAI-1-deficient mice are prostasin-dependent.

**Figure 4:**
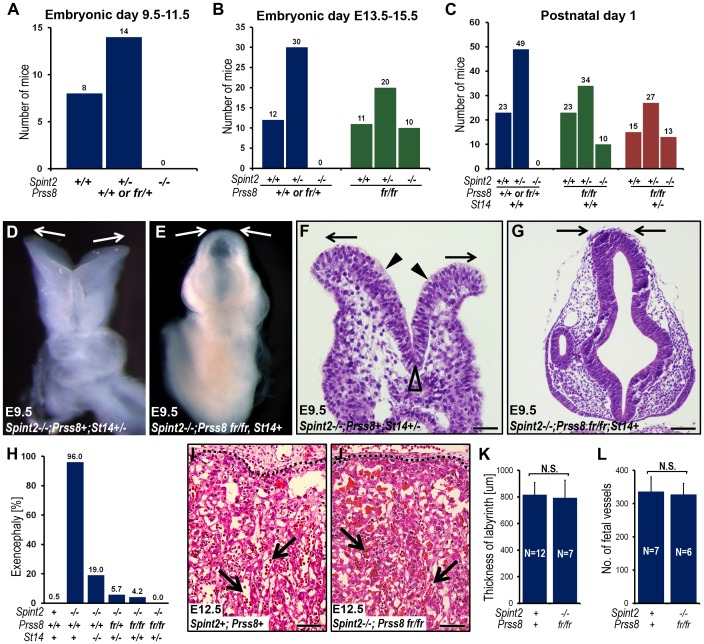
Reduced prostasin activity restores placental differentiation, embryonic survival, and neural tube closure in HAI-2–deficient mice. (A) Distribution of *Spint2* genotypes in prostasin-sufficient (*Prss8^+/+^* or *Prss8^+/fr^*) E9.5–11.5 offspring from interbred *Spint2* ^+/−^ *;Prss8^fr/+^* mice. No *Spint2^−/−^* embryos were observed (P<0.025, Chi-square test). (B) Distribution of *Spint2* genotypes in prostasin-sufficient (*Prss8^+/+^* or *Prss8^+/fr^*, blue bars) and prostasin-deficient (*Prss8^fr/fr^*, green bars) mouse embryos from interbred *Spint2* ^+/−^ *;Prss8^fr/+^* mice at E13.5–15.5. No prostasin-expressing *Spint2^−/−^* embryos were observed (P<0.001, Chi-square test), while survival of prostasin-deficient *Spint2^−/−^* embryos was restored. (C) Distribution of *Spint2* genotypes in newborn prostasin-sufficient, matriptase wildtype (*Prss8^+/+^* or *Prss8^+/fr^*;*St14^+/+^*, blue bars), prostasin-deficient, matriptase wildtype (*Prss8^fr/fr^;St14^+/+^*, green bars), and prostasin-deficient, matriptase haploinsufficient (*Prss8^fr/fr^;St14^+/−^*, red bars) offspring from *Spint2* ^+/−^ *;Prss8^fr/−^*×*Spint2* ^+/−^ *;Prss8^fr/+^;St14^+/−^* breeding pairs. Reduced prostasin activity restored embryonic survival of *Spint2^−/−^* mice partially in matriptase wildtype and completely in matriptase haploinsufficient mice. (D–G) Macroscopic (D and E) and histological (H&E staining) (F and G) appearance of the HAI-2-deficient, matriptase- and prostasin-sufficient (*Spint2^−/−^;Prss8^+/+^* or *Prss8^+/fr^*, *St14^+/−^*, D and F) or HAI-2- and prostasin-deficient, matriptase-sufficient (*Spint2^−/−^;Prss8^fr/fr^*, *St14^+/+^* or *St14^+/−^*) (E and G) embryos at E9.5. HAI-2 deficiency prevents convergence of neural folds in the cranial region of neural tube (D and F, arrows) leading to exencephaly. Convergence and fusion of neural folds are restored in HAI-2-deficient mice with low prostasin activity (E and G, arrows). Presence of medial (F, open arrowhead) and absence of dorsolateral (F, arrowheads) hinge points. (H) Frequency of exencephaly in E9.5–18.5 *Spint2^−/−^* embryos with different levels of prostasin activity (*Prss8^+/+^*, *Prss8^fr/+^* or *Psrr8^fr/fr^*) and matriptase (*St14^+/+^*, *St14^+/−^* or *St14^−/−^*). The frequency of neural tube defects is inversely correlated with the combined number of wildtype *Prss8* and *St14* alleles. A total of 524 embryos were analyzed. (I–L) Histological appearance (H&E staining) (I and J), thickness of placental labyrinth (K), and number of fetal vessels within the labyrinth (L) in the placentas of HAI-2 and prostasin-sufficient (*Spint2^+^;Prss8^+^*) and HAI-2 and prostasin double-deficient (*Spint2^−/−^;Prss8^fr/fr^*) embryos at E12.5. Reduced prostasin activity restores differentiation of placental labyrinth in *Spint2^−/−^* mice to levels not significantly (N.S.) different from wildtype littermate controls. Arrows in I and J show examples of fetal vessels. Scale bars: F, 50 µm G, I, and J, 100 µm.

To determine the impact of diminished prostasin activity on the developmental defects associated with HAI-2-deficiency, we next analyzed neural tube closure, placental differentiation, and overall survival of the offspring of interbred *Spint2^+/−^;Prss8^fr/+^* mice. Analysis of the genotype distribution of embryos at E9.5–11.5 did not identify any HAI-2-deficient embryos carrying at least one wildtype *Prss8* allele (*Spint2^−/−^;Prss8^+/+^* or *Spint2^−/−^;Prss8^fr/+^*) (Figure 4A). Interestingly, however, HAI-2-deficient embryos carrying two mutant *Prss8* alleles (*Spint2^−/−^;Prss8^−fr/fr^*) were found in the expected Mendelian ratio as late as E13.5–15.5 (Figure 4B, green bars). Furthermore, genotyping of newborn offspring revealed the presence of living *Spint2^−/−^;Prss8^fr/fr^* pups (Figure 4C, green bars), although they were found at slightly lower than expected frequency (15% vs. expected 25%, P<0.06, Chi-square test). These data strongly suggest that matriptase and prostasin act as part of a single proteolytic cascade to cause developmental defects in HAI-2-deficient mice. If this were the case, we hypothesized that lowering the activity of this cascade even further by eliminating one *St14* allele from *Spint2^−/−^;Prss8^fr/fr^* embryos should additionally improve the term survival of HAI-2-deficient mice. Indeed, genotyping of born offspring from interbred *Spint2^+/−^;Prss8^fr/+^;St14^+/−^* mice showed a normal distribution of *Spint2* alleles in *Prss8^fr/fr^;St14^+/−^* pups (Figure 4C, red bars), further suggesting that failure to regulate a proteolytic pathway including matriptase and prostasin accounts for all of the embryonic lethality caused by loss of HAI-2.

As reported previously, neural tube defects, including exencephaly, spina bifida, and curly tail were seen in 95–100% of *Spint2^−/−^;Prss8^fr/+^;St14^+/−^* mice ([43], this study). Examination of embryonic and extraembryonic tissues from *Spint2^−/−^;Prss8^fr/fr^* and *Spint2^−/−^;Prss8^fr/fr^;St14^+/−^* embryos, however, revealed that reduced prostasin activity sufficed to almost completely rescue the defects in both neural tube closure and placental differentiation caused by HAI-2 deficiency. Thus, macroscopic (Figure 4D and 4E) and histological (Figure 4F and 4G) examination of *Spint2^−/−^;Prss8^fr/fr^;St14^+/+^* and *Spint2^−/−^;Prss8^fr/fr^;St14^+/−^* embryos showed that, respectively, 5% and 0%, of these embryos exhibited exencephaly when analyzed after E9.5 (Figure 4H), and no embryos with either spina bifida or curly tail were observed (data not shown). Similarly, histological analysis of placental tissues from E10.5–E13.5 *Spint2^−/−^;Prss8^fr/fr^* or *Spint2^−/−^;Prss8^fr/fr^;St14^+/−^* embryos did not reveal any of the stereotypic defects associated with HAI-2 deficiency (Figure 4I and 4J). Thus, the overall appearance of the placental layers (Figure 4I and 4J), the thickness of the placental labyrinth (Figure 4K), and the number of fetal vessels within the labyrinth (Figure 4L), all were comparable to the HAI-2-sufficient littermate controls. In conclusion, these data document an essential role of prostasin in the etiology of all of the developmental defects previously observed in HAI-2-deficient mice.

### Prostasin is required for the activation of matriptase during development

**Figure 5:**
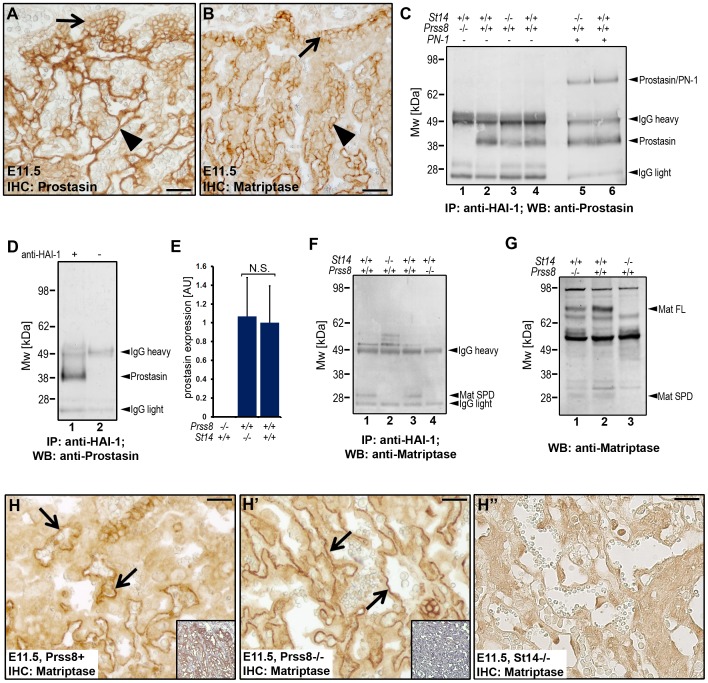
Prostasin is required for the activation of matriptase during placental differentiation. (A and B) Expression of prostasin (A) and matriptase (B) in placental tissues of wildtype mice at E11.5. Both proteins were expressed in the chorionic (arrows) and labyrinthine (arrowheads) trophoblasts. (C) Western blot detection of active prostasin in the fetal part of the placenta of wildtype (*Prss8^+/+^* and *St14^+/+^*, lanes 2, 4, and 6), prostasin-deficient (*St14^+/+^;Prss8^−/−^*) (lane 1), and matriptase-deficient (*St14^−/−^;Prss8^+/+^*) (lanes 3 and 5) embryos at E11.5 after immunoprecipitation with anti-mouse HAI-1 antibodies. Immunoprecipitated proteins in lanes 5 and 6 were acid-exposed to dissociate prostasin-HAI-1 complexes, and then incubated with PN-1 prior to western blot analysis. Positions of bands corresponding to active prostasin, prostasin/PN-1 complex, as well as non-specific signals of IgG heavy and light chains are indicated on the right. Positions of molecular weight markers (kDa) are shown on the left. (D) Omission of anti-HAI-1 antibody resulted in loss of detectable prostasin (compare lanes 1 and 2), indicating that the detected prostasin formed complexes with HAI-1. (E) Quantification of the relative amount of active prostasin in wildtype and matriptase placentae by densitometric scanning of prostasin western blots of HAI-1 immunoprecipitated material from (*Prss8^−/−^;St14^+/+^*, N = 3, *Prss8^+/+^;St14^−/−^*, N = 3, and *Prss8^+/+^;St14^+/+^*, N = 6). Data are shown as mean ± standard deviation (N.S., not significant). (F and G) Western blot detection of active matriptase in the fetal part of the placenta at E11.5 (F) after anti-HAI-1 immunoprecipitation, and in the epidermis of newborn skin (G) of wildtype (*Prss8^+/+^* and *St14^+/+^*) (F, lanes 1, and 3, and G, lane 2), prostasin-deficient (*St14^+/+^;Prss8^−/−^*) (F, lane 4 and G, lane 1), and matriptase-deficient (*St14^−/−^;Prss8^+/+^*) (F, lane 2, and G, lane 3) embryos. A 30 kDa band representing the active serine protease domain of matriptase (Mat SPD) was present in extracts from wildtype (lanes 1 and 3 in F), but not in matriptase- (lane 2 in F) or prostasin-deficient (lane 4 in F) placenta. Zymogen (Mat FL) and active (Mat SPD) forms of matriptase were detected in extracts from both wildtype and prostasin-deficient, but not matriptase-deficient epidermis. (H–H″) Immunohistochemical staining of matriptase in control *Prss8^+^* (H) and prostasin-deficient *Prss8^−/−^* (H′) placenta at E11.5. Specificity of staining of chorionic and labyrinthine trophoblasts (examples with arrows) is shown by the absence of staining of corresponding cells in *St14^−/−^* placenta (H″). Insets in H and H′ are parallel sections stained with prostasin antibodies. Open arrowheads in H–H″ show examples of non-specific staining. Scale bars: A, B, H, H′, and H″, 50 µm.

Matriptase was previously identified as an essential proteolytic activator of prostasin in the epidermis, and the near ubiquitous co-localization of the two membrane serine proteases in the epithelial compartment of most other adult tissues indicate that this matriptase-prostasin proteolytic pathway may be operating in multiple epithelia to maintain tissue homeostasis [23], [24], [25], [26]. The genetic epistasis analysis performed above provided strong evidence that matriptase and prostasin also are part of a single proteolytic cascade in the context of embryonic development. Furthermore, the striking overlap in expression of the two proteases documented earlier in the surface ectoderm during neural tube closure (see above) was also observed in the developing placenta (compare Figure 5A and 5B). To further investigate the functional interrelationship between the two proteases, we analyzed the levels of the activated forms of matriptase and prostasin in embryonic and placental tissues from matriptase- (*St14^−/−^*) or prostasin- (*Prss8^−/−^*) deficient mice at E11.5. Trypsin-like serine proteases are activated by autocatalytic or heterocatalytic cleavage after an arginine or lysine residue, located in a conserved activation motif within the catalytic domain. Activation cleavage severs the bond between the catalytic domain and upstream accessory domains, but the activated protease domain remains connected to upstream accessory domains by a disulfide bond [50]. Zymogen activation, therefore, can be detected by a mobility shift in reducing SDS-PAGE gels, which breaks the disulfide bond that keeps the two domains together. Direct detection of active matriptase in placental tissues by western blot, however, proved unsuccessful due to low signal intensity and a strong cross reactivity of available anti-matriptase antibodies with unrelated antigens. Similarly, direct detection of active prostasin by western blot failed due to the small difference in the electrophoretic mobility of the zymogen and the active form of the enzyme (data not shown). In order to circumvent these problems, we instead determined the amount of active matriptase and prostasin that formed inhibitor complexes with endogenous HAI-1 in embryonic tissues from wildtype, matriptase-, and prostasin-deficient embryos. Immunoprecipitation of protein extracts using anti-mouse HAI-1 antibodies followed by western blot with prostasin antibodies detected the presence of the 38 kDa band in the placentas of wildtype mice (Figure 5C, lanes 2 and 4) and matriptase-deficient mice (Figure 5C, lane 3). This band was not detected in placental extracts from prostasin-deficient embryos (Figure 5C, lane 1) or when anti-HAI-1 antibodies were omitted from the assay (Figure 5D, compare lanes 1 and 2), indicating that it represents the active form of prostasin released from an inhibitory complex with HAI-1. In support of this, when prostasin from either matriptase-deficient or littermate wildtype control placental tissues was released from the immunoprecipitated HAI-1-prostasin complexes by brief exposure to low pH, it was able to form SDS-stable complex with PN-1, which requires the catalytic activity of prostasin (Figure 5C, compare lane 3 with 5 and lane 4 with 6). Quantification of the amount of active prostasin in wildtype and prostasin-deficient placentae by densitometric scans of western blots showed that the loss of matriptase did not affect the amount of active prostasin (Figure 5E). Taken together, these data suggest that the developing placenta does not require matriptase for the activation of prostasin.

Detection of matriptase by western blot after immunoprecipitation with anti-HAI-1 antibodies revealed the presence of a 30 kDa band corresponding to the activated matriptase serine protease domain in wildtype placental tissues, but not matriptase-deficient placental tissues (Figure 5F, compare lanes 1 and 2). Surprisingly, however, the active form of matriptase was also absent in the extracts from prostasin-deficient placentae (Figure 5F, compare lanes 3 and 4). The absence of matriptase was observed in four independent experiments using placentae from a total of seven prostasin-deficient mice and their prostasin-sufficient littermate controls (Figure S1B and data not shown). As expected, analysis of skin extracts from prostasin-deficient newborn mice and wildtype littermate controls using the same western blot conditions clearly showed the presence of the active form of matriptase in both the control and prostasin-deficient mice (Figure 5G, compare lanes 1 and 2 with lane 3), demonstrating that differences in the functional relationship between the two proteases exist in different tissues. Immunohistochemistry of placentae from littermate control and prostasin-deficient embryos showed no obvious difference in levels or pattern of matriptase expression (compare Figure 5H and 5H′).

**Figure 6:**
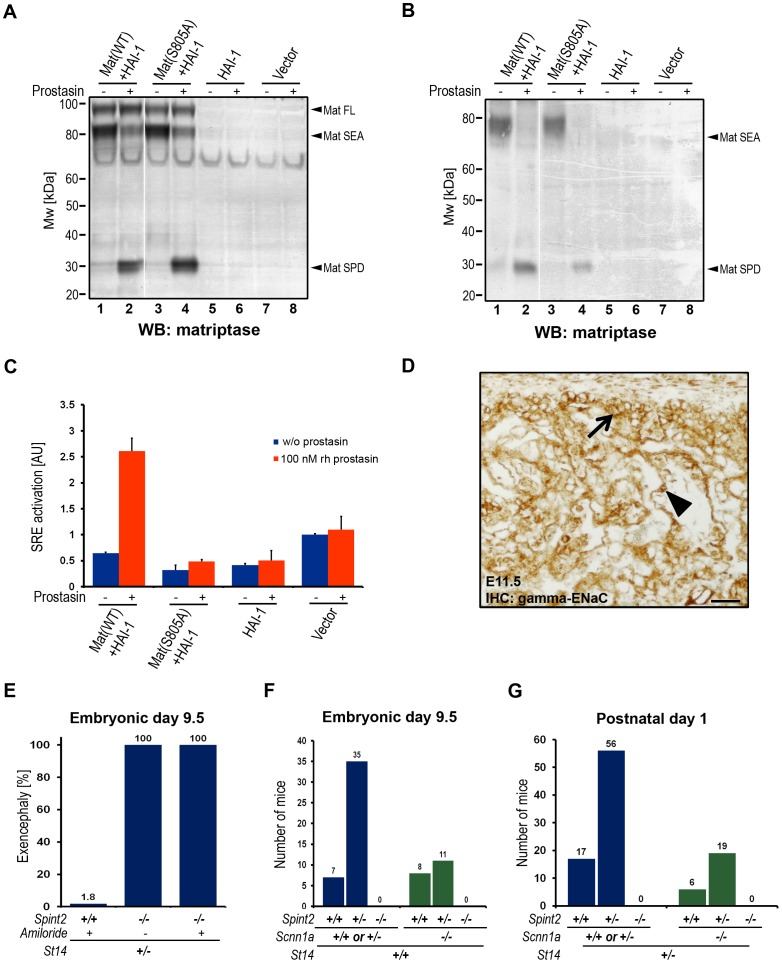
Prostasin activates matriptase on the surface of HEK293 cells. (A and B) Western blot detection of matriptase in cell lysates (A) and in the conditioned medium (B) from HEK293 cells transiently transfected with wildtype recombinant human matriptase and HAI-1 expression vectors (lanes 1 and 2), catalytically inactive (S805A) matriptase and HAI-1 (lanes 3 and 4), HAI-1 alone (lanes 5 and 6), and cells transfected with a control empty vector (lanes 7 and 8) that were incubated with (lanes 2, 4, 6, and 8) or without (lanes 1, 3, 5, and 7) 100 nM soluble recombinant human prostasin. Addition of prostasin promoted conversion of the matriptase zymogen to its activated two-chain form. Positions of bands corresponding to full length matriptase (Mat FL), matriptase pro-enzyme processed by autocatalytic cleavage within the SEA domain (Mat SEA), and activated matriptase serine protease domain (Mat SPD) are indicated on the right. Positions of molecular weight markers (kDa) are shown on left. (C) Quantification of the activation of PAR-2 in HEK293 cells expressing recombinant human PAR-2 in combination with wildtype (WT) or inactive (S805A) variants of matriptase and HAI-1, HAI-1 alone, or transfected with an empty vector, incubated without (blue bars) or with (red bars) 100 nM soluble recombinant human prostasin. Prostasin induced matriptase activity-dependent activation of PAR-2. (D) Immunohistochemical analysis of the expression of the gamma subunit of the epithelial sodium channel (ENaC) in placenta of control mice at E11.5. The expression was detected in the populations of chorionic (arrow) and labyrinthine (arrowhead) trophoblasts. Scale bar: 50 µm. (E) Frequency of exencephaly in amiloride-treated wildtype (*Spint2^+/+^*, N = 56), untreated HAI-2-deficient (*Spint2^−/−^*, N = 12) and amiloride-treated HAI-2-deficient (*Spint2^−/−^*, N = 7) embryos at E9.5. Amiloride treatment failed to rescue neural tube defects in *Spint2^−/−^; St14^+/−^* embryos. (F) Distribution of *Spint2* genotypes in ENaC-expressing (*Scnn1a^+/+^* or *Scnn1a^+/−^*, blue bars) and ENaC-deficient (*Scnn1a^−/−^*, green bars) offspring from *Spint2* ^+/−^ *;Scnn1a^+/−^*×*Spint2* ^+/−^ *;Scnn1a^+/−^* breeding pairs at E9.5. Loss of ENaC expression did not rescue early embryonic lethality in *Spint2^−/−^* mice. (G) Distribution of *Spint2* genotypes in matriptase-haploinsufficient ENaC-expressing (*St14^+/−^*;*Scnn1a^+/+^* or *Scnn1a^+/−^*, blue bars) and ENaC-deficient (*St14^+/−^*; *Scnn1a^−/−^*, green bars) offspring from *Spint2* ^+/−^ *;Scnn1a^+/−^, St14^+/−^*×*Spint2* ^+/−^ *;Scnn1a^+/−^;St14^+/+^* breeding pairs at birth. Loss of ENaC expression did not rescue overall embryonic survival in *Spint2^−/−^; St14^+/−^* mice.

To further substantiate the above findings, we next determined if prostasin could serve as an activator of matriptase in a reconstituted cell-based assay. For this purpose, we transiently transfected HEK-293 cells with expression vectors encoding HAI-1 (to allow for efficient matriptase expression) and wildtype or catalytically inactive matriptase. The transfected cells were then exposed to soluble recombinant prostasin or vehicle, and matriptase activation was analyzed six hours later by western blot of cell lysates (Figure 6A) or conditioned medium (Figure 6B). Interestingly, soluble prostasin efficiently activated matriptase, as evidenced by the large increase in the amount of the liberated matriptase serine protease domain (Mat SPD, Figure 6A and 6B, compare lanes 1 and 2) after reducing SDS/PAGE, and a corresponding diminution of the amount of matriptase zymogen (Mat SEA, Figure 6A and 6B, compare lanes 1 and 2). Activation site cleavage of matriptase by prostasin did not require matriptase catalytic activity, as shown by the increased amount of the isolated matriptase serine protease domain in prostasin-treated cells expressing a catalytically inactive matriptase (Figure 6A and 6B, compare lanes 3 and 4). Similar results were obtained when matriptase-transfected HEK-293 cells were transfected with a prostasin expression vector, rather than being treated with soluble prostasin (data not shown). To investigate if the prostasin-activated matriptase displayed functional activity, the HEK-293 cells described above were also transfected with a PAR-2 expression vector and a serum response element (SRE)-luciferase reporter plasmid to measure PAR-2 activity (Figure 6C). Exposure of serum-starved cells to soluble prostasin resulted in a large increase in luciferase activity in cells transfected with wildtype matriptase (Figure 6C, left panels), but not in cells transfected with catalytically inactive matriptase (Figure 6C, second panels from left), with HAI-1 alone (Figure 6C, second panels from right) or with empty vector (Figure 6C, right panels). Taken together, the data indicate that prostasin can proteolytically activate matriptase and is critical for the generation of active matriptase during placental development. Detection of active matriptase and prostasin in the embryo by western blot or by anti-HAI-1 immunoprecipitation failed to detect either of the proteases, likely due to the restricted expression of both proteins (data not shown).

### Developmental defects in HAI-2–deficient embryos are not caused by aberrant activity of the epithelial sodium channel

Both matriptase and prostasin have been reported to activate the epithelial sodium channel (ENaC) in cell-based assays, and prostasin is a critical regulator of ENaC activity during alveolar fluid clearance in mouse lungs and likely regulates ENaC activity in many other adult organs [51], [52]. Immunohistological analysis of embryonic tissues at E11.5 revealed strong expression of ENaC in the developing labyrinth layer of the placenta (Figure 6D). No ENaC expression was detected in the embryo proper (data not shown). To investigate a possible involvement of ENaC in the etiology of prostasin-matriptase-induced developmental defects in HAI-2-deficient mice, pregnant females from *Spint2^+/−^* mice bred to *Spint2^+/−^;St14^+/−^* mice were treated daily between E5.5–8.5 with the pharmacological inhibitor of ENaC activity, amiloride, which is known to cross the feto-maternal barrier [53]. Genotyping of embryos extracted at E9.5 from these crosses did not identify any *Spint2^−/−^;St14^+/+^* embryos (Figure S1C), indicating that the inhibition of ENaC activity is not sufficient to prevent early embryonic lethality resulting from the loss of HAI-2. Furthermore, all of the seven *Spint2^−/−^;St14^+/−^* embryos identified in this experiment exhibited exencephaly, suggesting that ENaC activity is not critically involved in the etiology of neural tube defects in HAI-2-deficient mice (Figure 6E). Similarly, genetic inactivation of the α subunit of ENaC (encoded by *Scnn1a*), which is necessary for channel activity *in vivo* [54], [55], failed to rescue embryonic development of HAI-2-deficient animals, as evidenced by a complete absence of any surviving *Spint2^−/−^;Scnn1a^−/−^* double-deficient embryos at E9.5 from *Spint2^+/−^;Scnn1a^+/−^* mice bred to *Spint2^+/−^;Scnn1a^+/−^* mice (Figure 6F) and the failure of *Spint2^−/−^;Scnn1a^−/−^* double-deficient mice to appear in the newborn offspring from *Spint2^+/−^;Scnn1a^+/−^* mice bred to *Spint2^+/−^;Scnn1a^+/−^;St14^+/−^* mice (Figure 6G). Taken together, these data do not support the critical involvement of aberrant ENaC activity in the developmental defects resulting from lack of HAI-2 regulation of the prostasin-matriptase proteolytic pathway.

### Excess PAR-2 signaling does not cause developmental defects in HAI-2–deficient mice

**Figure 7:**
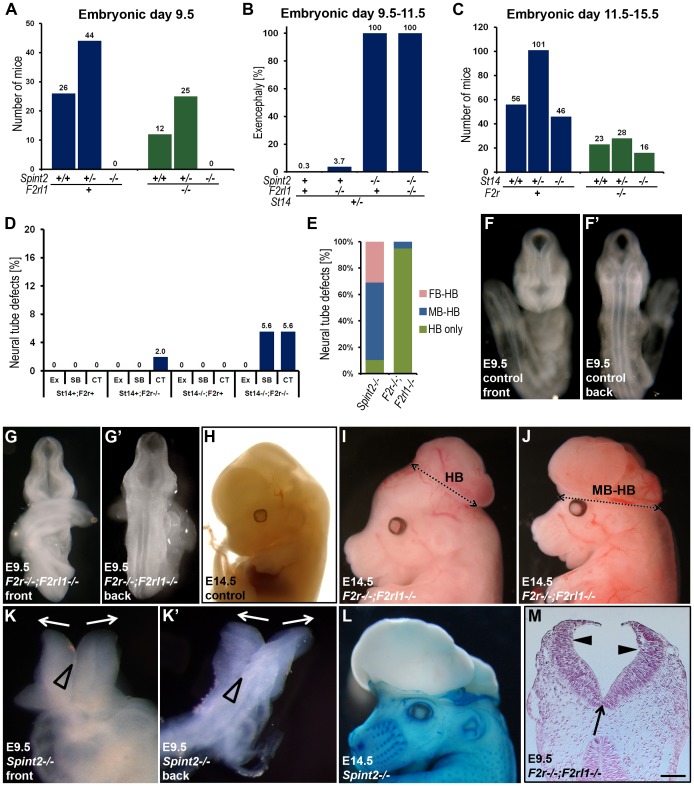
Neural tube defects and embryonic lethality in HAI-2–deficient mice are not dependent on PAR-2, and combined PAR-1 and matriptase deficiency does not phenocopy combined PAR-1 and PAR-2 deficiency. (A) Distribution of *Spint2* genotypes at E9.5 in PAR-2-expressing (*F2rl1^+/+^* or *F2rl1^+/−^*, blue bars) and PAR-2-deficient (*F2rl1^−/−^*, green bars) offspring from interbred *Spint2^+/−^,F2rl1^+/−^* mice. No *Spint2^−/−^* embryos were detected irrespective of PAR-2 expression. (B) Frequency of exencephaly observed in HAI-2 and PAR-2-sufficient (*Spint2^+^;F2rl1^+^* N = 366), PAR-2-deficient (*Spint2^+^;F2rl1^−/−^*, N = 164), HAI-2-deficient (*Spint2^−/−^,F2rl1^+^*, N = 18), and PAR-2 and HAI-2 double- (*Spint2^−/−^;F2rl1^−/−^*, N = 12) deficient embryos extracted at E9.5–E11.5. Loss of PAR-2 activity fails to correct neural tube defects in HAI-2-deficient embryos. (C) Distribution of *St14* alleles at E11.5–15.5 in PAR-1-expressing (*F2r^+/+^* or *F2r^+/−^*, blue bars) and PAR-1-deficient (*F2r^−/−^*, green bars) embryos from interbred *St14* ^+/−^ *;F2r^+/−^* mice. Loss of PAR-1 activity does not affect embryonic survival of matriptase-deficient mice. (D) Frequency of exencephaly (Ex), spina bifida (SB), and curly tail (CT) in E9.5–18.5 embryos with different levels of expression of PAR-1 (*F2r^+^* or *F2r^−/−^*) and matriptase (*St14^+^*or *St14^−/−^*). A total of 326 embryos were analyzed. Loss of matriptase does not significantly increase the incidence of neural tube defects in PAR-1-deficient embryos. (E) Comparison of the severity of exencephaly in HAI-2-deficient (*Spint2^−/−^*, N = 29) and PAR-1 and PAR-2 double-deficient (*F2r^−/−^;F2rl1^−/−^*, N = 39) embryos. 95% of affected *F2r^−/−^;F2rl1^−/−^* embryos exhibited exencephaly that was confined to hindbrain region of the cranium (HB only, green bars), with the remaining 5% extending to the midbrain region (MB-HB, blue bars). In contrast, only 10% of exencephalies observed in *Spint2^−/−^*-deficient mice were confined to the hindbrain, with 59% extended to midbrain, and 31% to forebrain region (FB-HB, red bars). (F–G′) Ventral (F and G) and dorsal (F′ and G′) view of non-affected control (F and F′) and affected PAR-1 and PAR-2 double-deficient (*F2r^−/−^;F2rl1^−/−^*) (G and G′) embryos at E9.5. The initial stages of neural tube closure all appear to be unaffected by the combined absence of PAR-1 and PAR-2. (H–J) Appearance of control (H) and PAR-1 and PAR-2 double-deficient embryos with exencephaly (I and J) at E14.5. Exencephaly in 95% of the affected PAR-1 and PAR-2 double-deficient embryos was restricted to hindbrain region (HB, two-sided arrow in I) and extended to midbrain (MB-HB, two-sided arrow in J) in only 5% of the cases. (K and K′) Ventral (K) and dorsal (K′) view of the macroscopic appearance of HAI-2-deficient (*Spint2^−/−^*) embryos at E9.5. Divergence of neural folds (arrows) and defects in neural tube closure extending from forebrain region to cervix are obvious. Open arrowheads show normal formation of medial hinge points. (L) Macroscopic appearance of a HAI-2-deficient embryo with exencephaly at E14.5. 90% of embryos presented with exencephaly that included at least midbrain and hindbrain regions of the developing cranium. (M) Histological appearance (nuclear fast red staining) of PAR-1 and PAR-2 double-deficient embryo with exencephaly at E9.5. Defined medial (arrow) and dorsolateral (arrowheads) hinge points are clearly visible. Scale bar: 150 µm.

Matriptase and prostasin are co-expressed with PAR-2 in surface ectoderm during neural tube closure ([43], [45], this study), and matriptase displays extraordinarily favorable activation kinetics towards PAR-2 in cell-based assays [43], [45]. Furthermore, activation of PAR-2 (encoded by the *F2rl1* gene) was recently shown to contribute to neural tube closure (see below). These data suggested that some, or all, of the prostasin- and matriptase-dependent defects in HAI-2-deficient mice could be caused by excess PAR-2 signaling. To test this hypothesis, we interbred *Spint2^+/−^*;*F2rl1^+/−^* mice and genotyped the ensuing embryos at E9.5. This analysis failed to identify any *Spint2^−/−^;F2rl1^−/−^* embryos (Figure 7A). Thus, the loss of PAR-2 activity is not sufficient to overcome matriptase- and prostasin-dependent early embryonic lethality in HAI-2-deficient mice. When the early embryonic survival was improved by matriptase haploinsufficiency (see above), analysis of neural tubes at E9.5 revealed exencephaly in 100% of *Spint2^−/−^;F2rl1^−/−^ St14^+/−^* embryos, identical to the frequency of defects observed in littermate HAI-2-deficient embryos expressing PAR-2 (*Spint2^−/−^;F2rl1^+/+^* or *F2rl1^+/−^;St14^+/−^*) (Figure 7B). Thus, excess PAR-2 activation does not appear to be critically involved in the etiology of neural tube defects in HAI-2-deficient mice.

### Loss of matriptase does not cause neural tube defects in PAR-1–deficient embryos

Rac1 activation in surface ectoderm through G_i_, initiated by either PAR-1 or PAR-2 activation was recently shown to be required for neural tube closure. Thus, mice with combined, but not single, deficiency in PAR-1 and PAR-2 display exencephaly with high frequency [45]. As matriptase and prostasin are co-expressed with PAR-2 in surface ectoderm during neural tube closure ([43], [45], this study), we next investigated if the prostasin-matriptase cascade identified in the current study contributes to physiological PAR-2 activation during neural tube closure. To test this, we generated mice with combined deficiency in PAR-1 (encoded by the *F2r* gene) and matriptase (*F2r^−/−^;St14^−/−^*). If matriptase was essential for the activation of PAR-2 during neural tube closure, these mice should phenocopy mice with a combined PAR-1 and PAR-2 deficiency, including embryonic lethality and high susceptibility to cranial neural tube defects [45]. However, analysis of the midgestation embryos from intercrossed *F2r^+/−^;St14^+/−^* mice yielded the expected distribution of all genotypes (Figure 7C). Furthermore, none of 20 observed *F2r^−/−^;St14^−/−^* mice displayed cranial neural tube defects, although the PAR-1 deficiency alone or in combination with matriptase deficiency occasionally led to defects in the closure of the posterior neural tube, resulting in spina bifida and curly tail (Figure 7D).

### HAI-2 and PAR-1/PAR-2 regulate different stages of neural tube development

The lack of functional interaction between prostasin-matriptase and PAR-1/PAR-2 regulated signaling pathways evidenced from the above experiments suggested that the two pathways are either involved in two essential, non-redundant mechanisms regulating the same steps of neural tube closure, or that they may regulate different stages of the process. To distinguish between the two possibilities, we performed a detailed morphologic comparison of the neural tube defects caused by loss of HAI-2 and by the combined loss of PAR-1 and PAR-2. Macroscopic analysis showed significant differences in the types of neural tube defects in the two mutant mouse strains. In PAR-1 and PAR-2 double-deficient mice, the defects were almost exclusively restricted to the hindbrain region of the cranial neural tube (Figure 7E and Table 2). In addition, PAR-1 and PAR-2 double-deficient embryos did not exhibit any obvious abnormalities during the early stages of neural tube closure, as all of the embryos analyzed before E10.5 showed normal elevation and conversion of the opposing neural folds, as well as the completion of the neural fold fusion at initial closure points 1 and 2 at hindbrain/cervix and forebrain/midbrain boundaries, respectively (compare Figure 7F with 7G, and 7F′ with 7G′). As a result, the neural tube defects in these mice were generally only obvious after E10.5 (compare Figure 7H with 7I and 7J), and were generally restricted to the hindbrain region of the neural tube, with less than five percent exhibiting exencephaly that extended to the midbrain region (Figure 7I and Table 2). In contrast, HAI-2 deficiency was generally associated with a failure of cranial neural tube closure that was obvious at E9.5 or earlier, and was due to the inability of neural folds to elevate properly, and to come into juxtaposition necessary for the fusion (compare Figure 7F with 7K, and 7F′ with 7K′). The fusion at closure point 1 of HAI-2-deficient mice was completed in all embryos analyzed at E9.5 or later, and no case of craniorachischisis was observed (Table 2). However, 10 percent of *Spint2^−/−^* embryos failed to initiate fusion at closure point 2, resulting in exencephaly that extended from forebrain region to the hindbrain-cervical boundary (Figure 7K and 7K′). In addition, even in the embryos that successfully initiated the fusion at closure point 2, the exencephaly was more extensive than the one observed in PAR-1 and PAR-2 double-deficient embryos, typically spanning the entire midbrain and hindbrain regions (compare Figure 7L and 7I). Finally, histological analysis of affected embryos at E9.5 showed that dorsolateral hinge points (DLHPs) critical for the final stages of the neural tube closure were absent in 97 percent of *Spint2^−/−^* embryos (Figure 4D and 4F, Figure 7K and 7K′, and Table 2), while *F2r^−/−^;F2rl1* ^−/−^ embryos generally exhibited DLHP formation indistinguishable from wildtype littermate controls (Figure 7G, 7G′, and 7M, compare to Figure 4E, 4G, Figure 7F and 7F′). Thus, substantial differences are observed in the location, frequency, extent, and onset of the neural tube defects of HAI-2-deficient mice and PAR-1 and PAR-2 double-deficient mice, further indicating the independent roles of, respectively, repression and activation of the two protease-regulated pathways in distinct stages of neural tube formation.

**Table 2:** Comparison of morphologic features of neural tube defects observed in *Spint2^−/^* ^−^ and *F2r^−/−^; F2rl1^−/−^* mice.

	*Spint2^−/−^*	*F2r^−/−^; F2rl1^−/−^*
**Process**		
Formation of medial hinge point	100% (28/28)	100% (38/38)
Formation of dorsolateral hinge points	3% (1/31)	97% (28/29)
Completion of C1 fusion	100% (28/28)	100% (66/66)
Completion of C2 fusion	90% (27/30)	100% (66/66)
**Extent of neural tube defect**		
Hindbrain only	10% (3/29)	95% (37/39)
Hindbrain and midbrain	59% (17/29)	5% (2/39)
Forebrain to cervix	31% (9/29)	0% (0/39)
Craniorachischisis	0% (0/29)	0% (0/39)

## Discussion

In this study, we exploited the uniform matriptase-dependent embryonic lethality of mice deficient in hepatocyte growth factor activator inhibitors as a means to genetically identify novel molecules and pathways regulating and being regulated by matriptase in the developing embryo by epistasis analysis. This analysis resulted in a number of unexpected findings. First, we found that prostasin is an essential component of the matriptase-dependent molecular machinery that causes early embryonic lethality, derails placental labyrinth formation, and causes defects in neural tube closure in these mice. This shows that both proteins are expressed, are active, functionally interact, and must be regulated by hepatocyte growth factor activator inhibitors already during early development. Surprisingly, however, rather than being a downstream effector of matriptase function, as previously established for both mouse and human epidermis ([23], [24], [25], [26], this study), prostasin acts upstream of matriptase during embryogenesis and is essential for activation of the matriptase zymogen. This finding is perplexing, as matriptase is well-established to be able to auto-activate, as most clearly evidenced by the inability of recombinant matriptase protein with the catalytic triad serine mutated to alanine (S805A matriptase) to undergo activation site cleavage [56]. Furthermore, prostasin shows no catalytic activity towards peptide sequences derived from the prostasin pro-peptide [57] and no reports of prostasin auto-activation have appeared to date. Substantiating this finding, however, we found that prostasin efficiently activated the matriptase zymogen in a reconstituted cell-based assay. These findings are aligned with a recent study showing that PAR-2 activation in some cultured cells, caused by exposure of cultured cells to exogenously added activated prostasin, was blunted by a neutralizing antibody directed against matriptase [45], providing further evidence that complex and context-specific relationships between the two membrane-anchored serine proteases may exist *in vivo*. Another important finding relating to the developmental prostasin-matriptase cascade identified in this study emanated from our biochemical analysis of placental tissues, which revealed that activated forms of both matriptase and prostasin were present in a complex with HAI-1 in placental tissues. This indicates that the regulation of the prostasin-matriptase cascade by HAI-1 (and likely HAI-2) may occur by controlling both prostasin and matriptase proteolytic activity. Furthermore, as both HAI-1 and HAI-2 are very promiscuous and display potent inhibitory activity towards a number of trypsin-like serine proteases *in vitro* [58], [59], [60], [61], [62], [63], [64], it is throughout plausible that they may also regulate the activity of as yet unidentified proteases that act upstream of, downstream of or between prostasin and matriptase. Such profound complexities in zymogen activation relationships between trypsin-like serine proteases and for the promiscuity of their cognate inhibitors have long been recognized in the coagulation, fibrinolytic, complement, and digestive systems. The current findings, thus, serve to underscore that our knowledge of the molecular workings of membrane-anchored serine proteases is still fragmentary, due to their quite recent emergence as a protease subfamily.

The outcome of our epistasis analysis querying the contribution of proHGF, PAR-2, and ENaC to the prostasin and matriptase-dependent embryonic demise of HAI-1- and HAI-2-deficient mice also was unanticipated. Each of the three proteins has been genetically validated as a substrate for either matriptase or prostasin in developmental or post-developmental processes, has established functions in embryonic development, and is developmentally co-expressed with both proteases. Nevertheless, their genetic elimination failed to prevent or alleviate any of the abnormalities caused by the loss of HAI-1 or HAI-2. Importantly, our analysis does not exclude that cleavage of either of the three proteins must be suppressed by HAI-1 or HAI-2 at later stages of development that cannot be analyzed by the current experimental approach. Also, the possibility that the lethality of HAI-1- or HAI-2-deficient embryos is caused by the simultaneous cleavage of more than one of these substrates cannot be formally excluded. It was particularly surprising that the neural tube defects associated with HAI-2-deficiency were unrelated to either excessive or reduced (through desensitization) PAR-2 activity, despite the unequivocal contribution of PAR-2 signaling to neural tube closure, and the wealth of strong circumstantial evidence that prostasin and matriptase contribute to PAR-2 activation in this process [45], [65]. Equally surprising in this regard, the combined loss of PAR-1 and matriptase failed to cause the neural tube closure defects observed in PAR-1 and PAR-2 double-deficient embryos, showing that matriptase is not essential for initiation of physiological PAR-2 signaling during neural tube formation. Previous analysis has identified five other membrane-anchored serine proteases and fourteen secreted trypsin-like serine proteases that are expressed during neural tube formation, some of which can activate PAR-2 in cell-based assays [43], [45]. It is therefore possible that the prostasin-matriptase cascade does contribute to PAR-2 activation during neural tube closure, but sufficient residual activation of PAR-2 by other developmentally co-expressed serine proteases takes place in its absence to allow for completion of this developmental process. Nevertheless, the careful comparison of the morphology of neural tube defects in PAR-1 and PAR-2, and HAI-2-double deficient embryos performed here revealed distinct differences in terms of their anatomical location and the stage of developmental failure. Taken together, these data suggest that promotion of neural tube closure by HAI-2 suppression of the prostasin-matriptase cascade and promotion of neural tube closure by PAR-1/PAR-2 signaling may be temporally and spatially distinct morphogenic processes.

In conclusion, this study identifies a prostasin-matriptase cell surface protease cascade whose activity must be suppressed by HAI-1 and HAI-2 to enable early embryonic ectoderm formation, placental morphogenesis, and neural tube closure.

## Materials and Methods

### Mouse strains

All experiments were performed in an Association for Assessment and Accreditation of Laboratory Animal Care International-accredited vivarium following Standard Operating Procedures. The studies were approved by the NIDCR Institutional Animal Care and Use Committee. All studies were littermate controlled. *Spint1^−/−^*, *Spint2^−/−^*, *St14^−/−^*, Hgfr*^−/−^*, *F2r^−/−^*, *F2rl1^−/−^*, *Scnn1a^−/−^*, and *Prss8^fr/fr^* mice have been described [38], [40], [43], [49], [66], [67], [68]. Prostasin-deficient (*Prss8^−/−^*) mice were generated by standard blastocyst injection of C57BL/6J-derived embryonic stem cells carrying a gene trap insertion in the *Prss8* gene (clone IST10122F12, Texas A&M Institute for Genomic Research, College Station, TX).

### Extraction of embryonic and perinatal tissues

Breeding females were checked for vaginal plugs in the morning and the day on which the plug was found was defined as the first day of pregnancy (E0.5). Pregnant females were euthanized in the mid-day at designated time points by CO_2_ asphyxiation. Embryos were extracted by Caesarian section and the individual embryos and placentae were dissected and processed. Visceral yolk sacs of individual embryos were washed twice in phosphate buffered saline, subjected to genomic DNA extraction and genotyped by PCR (see Table S1 for primer sequences). Newborn pups were euthanized by CO_2_ inhalation at 0°C. For histological analysis, the embryos and newborn pups were fixed for 18–20 hrs in 4% paraformaldehyde (PFA) in PBS, processed into paraffin, sectioned, and stained with hematoxylin and eosin (H&E), or used for immunohistochemistry as described below. For histomorphometric analysis of placental labyrinth, the midline cross sections of plancetal tissues were stained with H&E and the thickness of the labyrinth was determined as the maximum perpendicular distance of fetal vessel from the chorionic trophoblast layer.

### Immunohistochemistry

Antigens from 5 µm paraffin sections were retrieved by incubation for 10 min at 37°C with 10 µg/ml proteinase K (Fermentas, Hanover, MD) for HAI-1 staining, or by incubation for 20 min at 100°C in 0.01 M sodium citrate buffer, pH 6.0, for all other antigens. The sections were blocked with 2% bovine serum albumin in PBS, and incubated overnight at 4°C with rabbit anti-human CD31 (1∶100, Santa Cruz Biotechnology, Santa Cruz, CA), goat anti-mouse HAI-1 (1∶200, R&D Systems, Minneapolis, MN), mouse anti-human prostasin (1∶200, BD Transduction Laboratories, San Jose, CA), sheep anti-human matriptase (1∶200, R&D Systems) or ENaCγ subunit (1∶100, Sigma-Aldrich, St. Louis, MO) primary antibodies. Bound antibodies were visualized using biotin-conjugated anti-mouse, -rabbit, -sheep or -goat secondary antibodies (all 1∶400, Vector Laboratories, Burlingame, CA) and a Vectastain ABC kit (Vector Laboratories) using 3,3′-diaminobenzidine as the substrate (Sigma-Aldrich). All microscopic images were acquired on an Olympus BX40 microscope using an Olympus DP70 digital camera system (Olympus, Melville, NY).

### Protein extraction from mouse tissues

Placentae were extracted from embryos at E10.5 or E11.5. The embryonic portion of each placenta was manually separated from maternal decidua using a dissecting microscope. The tissues were then homogenized in ice-cold 50 mM Tris/HCl, pH 8.0; 1% NP-40; 500 mM NaCl buffer and incubated on ice for 10 minutes. The lysates were centrifuged at 20,000 g for 10 min at 4°C to remove the tissue debris and the supernatant was used for further analysis as described below.

### Detection of active matriptase and prostasin in mouse embryonic tissues

Lysates from two placentae of the same genotype were combined and pre-incubated with 100 ul GammaBind G Sepharose beads (GE Healthcare Bio-Sciences, Uppsala, Sweden) for 30 minutes at 4°C with gentle agitation. The samples were spun at 5,000 g for 1 min to remove the beads, and the supernatant was then incubated with 5 µg goat anti-mouse HAI-1 antibody (R&D Systems) and 100 ul of GammaBind G Sepharose beads for 3 hours at 4°C. The samples were spun at 5,000 g for 1 min, the supernatant was removed, and the beads were washed 3 times with 1 ml ice-cold 50 mM Tris/HCl, pH 8.0; 1% NP-40; 500 mM NaCl buffer. The beads were then mixed with 30 ul of 1× SDS loading buffer (Invitrogen, Carlsbad, CA) with 0.25 M β-mercaptoethanol, incubated for 5 min at 99°C, and cooled on ice for 2 minutes. The samples were spun at 5,000 g for 1 min and the released proteins were resolved by SDS-PAGE (4–12% polyacrylamide gel) and analyzed by western blot using mouse anti-human prostasin (1∶250, BD Transduction Labs) or sheep anti-human matriptase (1∶500, R&D Systems) primary antibodies and goat anti-mouse (DakoCytomation) or donkey anti-sheep (Sigma-Aldrich) secondary antibodies (both 1∶1000) conjugated to alkaline phosphatase, and visualized using nitro-blue tetrazolium and 5-bromo-4-chloro-3′-indolyphosphate.

### Amiloride injections

Five ug per g of body weight of amiloride (Sigma-Aldrich) in 10% DMSO in PBS was administered to pregnant females by intraperitoneal injection every 24 hours starting on E5.5. Embryos were extracted on E9.5 by Caesarian section and genotyped as described, and scored for neural tube closure defects.

### Generation of soluble recombinant wildtype, catalytically inactive S238A, and V170D prostasin zymogens

The generation of pIRES2-EGFP-prostasin has been described [26]. Substitution of the native prostasin activation site (APQAR) by the enteropeptidase-dependent cleavage site (DDDDK), and either the S238A or V170D point mutations were introduced using the QuickChange Kit (Stratagene, La Jolla, CA) and the following primers, respectively: 5′-GCTCCCTGCGGTGTGGCCCCCCAAGCACGCATCACAGGTGGCAGC-3′, 5′-GACGCCTGCCAGGGTGACGCTGGGGGCCCACTCTCCTGC-3′, and 5′-GGCCTCCACTGCACTGACACTGGCTGGGGTCAT-3′. Successful mutagenesis was verified by sequencing of both strands of the resulting cDNA. Expression plasmids carrying individual mutations were transiently transfected into HEK-293T cells using Turbofect (Fermentas). The cells were grown for two days and soluble recombinant prostasin was prepared by treatment of cells with phosphatidylinositol-specific phospholipase C (Sigma-Aldrich) as described previously [26].

### Determination of enzymatic activity of recombinant prostasin variants

Recombinant wildtype, V170D Frizzy or catalytically inactive S238A prostasin zymogen variants were first incubated with 5.1 U recombinant bovine enteropeptidase (Novagen, Cambridge, MA) overnight at 37°C in enterokinase buffer (Novagen). Following enteropeptidase removal using the Enterokinase Removal Kit (Sigma-Aldrich), the protein concentration was estimated by western blot of serially diluted proteins using a reference with known protein concentration. For substrate hydrolysis assays, the activated prostasin variants (62.5 nM) were incubated with the fluorogenic substrate pERTKR-AMC (50 µM final concentration) (R&D systems) at 37°C in 50 mM NaCl, 50 mM Tris-HCl pH 8.8, 0.01% Tween-20 buffer, and the fluorescence was measured using a Wallac plate reader (Perkin Elmer, Waltham, MA). Each measurement was performed in triplicate. For serpin complex formation, prostasin variants were diluted in 50 mM Tris-HCl, pH 9.0, 50 mM NaCl, 0.01% Tween 20 to a final concentration of 150 nM, incubated with 250 ng recombinant human protease nexin-1 (PN-1) (R&D Systems) for 1 h at 37°C, and analyzed using 12% reducing SDS-PAGE and western blotting, using a monoclonal anti-prostasin antibody (BD Transduction Laboratories).

### Matriptase activation and SRE–luciferase assay

HEK 293 cells were plated in 24-well plates and grown in DMEM supplemented with 10% FBS for 24 h. Cells were co-transfected with pSRE-firefly luciferase (50 ng), pRL-Renilla luciferase (20 ng), pcDNA 3.1 Par2 (100 ng) (Missouri S&T cDNA Resource Center) using Lipofectamine and Plus reagent (Invitrogen), pcDNA 3.1 expression vectors containing wildtype human matriptase or catalytically dead matriptase (S805A), full length human HAI-1 [69] and empty pcDNA 3.1 vector to equalize the total amount of transfected DNA. After 36 h the cells were serum starved over night and then stimulated with 100 nM recombinant human soluble prostasin (R&D) or vehicle for 6 h. Cell were lysed and luciferase activity was determined using the dual luciferase assay kit (Promega, Madison, WI) according to the manufacturer's instructions. Chemiluminiscence was measured using Microtiter Plate Luminometer (Dynex Technologies, Chantilly, VA) and the SRE activation was determined as the ratio of firefly to Renilla luciferase counts. The assay was performed two times in duplicates.

## Supporting Information

Figure S1 (A) Western blot detection of protein nexin-1 (PN-1). Wildtype zymogen (lanes 1 and 2), activated wildtype (lanes 3 and 4), V170D (frizzy) zymogen (lanes 5 and 6), activated V170D (lanes 7 and 8), S238A zymogen (lanes 9 and 10), and activated S238A (lanes 11 and 12) prostasin variants were incubated with (lanes 2, 4, 6, 8, 10, and 12) or without (lanes 1, 3, 5, 7, 9, and 11) 250 ng of recombinant human PN-1. Position of PN-1, and predicted position of prostasin/PN-1 complexes (not detected by anti-PN-1 antibody presumably due to significant molecular rearrangement of PN-1 in the complex with the protease) are indicated. Positions of molecular weight markers (kDa) are shown on left. (B) Western blot detection of active matriptase in the fetal part of the E11.5 placentas of one matriptase-deficient (*St14^−/−^;Prss8^+/+^*) (lane 1), three wildtype (*Prss8^+/+^* and *St14^+/+^*) (lanes 2,3, and 4), and three prostasin-deficient (*St14^+/+^;Prss8^−/−^*) (lanes 5, 6, and 7) embryos after anti-HAI-1 immunoprecipitation. A 30 kDa band representing the active serine protease domain of matriptase (Mat SPD) was present in extracts from wildtype, but not in matriptase- or prostasin-deficient placentas. (C) Distribution of *Spint2* genotypes at E9.5 in offspring from interbred *Spint2* ^+/−^ breeding pairs treated with the ENaC inhibitor, amiloride, at E5.5–8.5. No *Spint2^−/−^* embryos were observed.
(TIF)

Table S1 Sequences of PCR primers used for mouse genotyping.
(DOCX)
