# Supplementary figures and images for: Reduced Prostasin (CAP1/PRSS8) Activity Eliminates HAI-1 and HAI-2 Deficiency–Associated Developmental Defects by Preventing Matriptase Activation
Source: PLoS Genet. 2012 Aug 30;8(8):e1002937. doi: 10.1371/journal.pgen.1002937 (PMC3431340; doi:10.1371/journal.pgen.1002937)

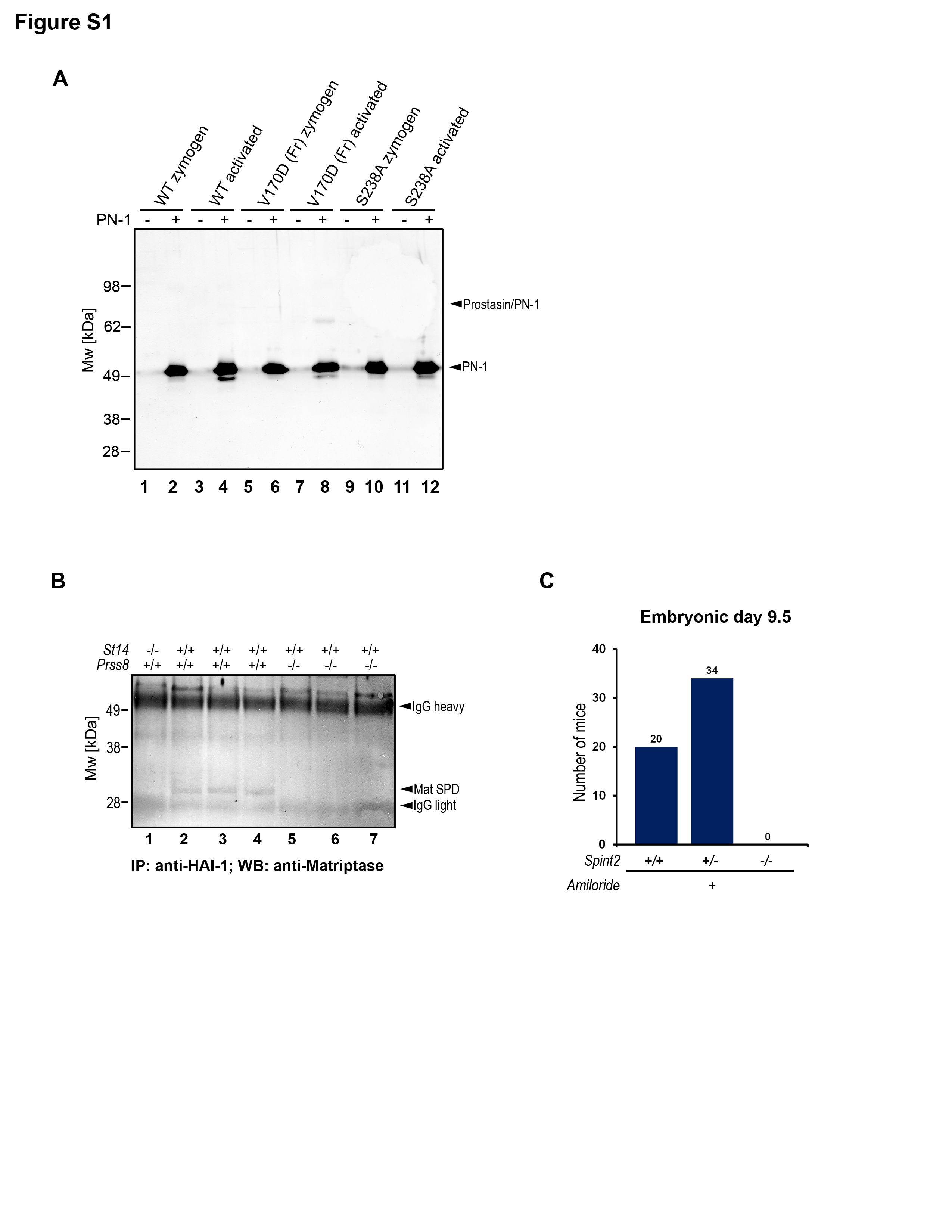

Supplement: Figure S1 — (A) Western blot detection of protein nexin-1 (PN-1). Wildtype zymogen (lanes 1 and 2), activated wildtype (lanes 3 and 4), V170D (frizzy) zymogen (lanes 5 and 6), activated V170D (lanes 7 and 8), S238A zymogen (lanes 9 and 10), and activated S238A (lanes 11 and 12) prostasin variants were incubated with (lanes 2, 4, 6, 8, 10, and 12) or without (lanes 1, 3, 5, 7, 9, and 11) 250 ng of recombinant human PN-1. Position of PN-1, and predicted position of prostasin/PN-1 complexes (not detected by anti-PN-1 antibody presumably due to significant molecular rearrangement of PN-1 in the complex with the protease) are indicated. Positions of molecular weight markers (kDa) are shown on left. (B) Western blot detection of active matriptase in the fetal part of the E11.5 placentas of one matriptase-deficient (St14−/−;Prss8+/+) (lane 1), three wildtype (Prss8+/+ and St14+/+) (lanes 2,3, and 4), and three prostasin-deficient (St14+/+;Prss8−/−) (lanes 5, 6, and 7) embryos after anti-HAI-1 immunoprecipitation. A 30 kDa band representing the active serine protease domain of matriptase (Mat SPD) was present in extracts from wildtype, but not in matriptase- or prostasin-deficient placentas. (C) Distribution of Spint2 genotypes at E9.5 in offspring from interbred Spint2 +/− breeding pairs treated with the ENaC inhibitor, amiloride, at E5.5–8.5. No Spint2−/− embryos were observed. (TIF) [file pgen.1002937.s001.tif]
