# Supplementary material for: Reduced Prostasin (CAP1/PRSS8) Activity Eliminates HAI-1 and HAI-2 Deficiency–Associated Developmental Defects by Preventing Matriptase Activation
Source: PLoS Genet. 2012 Aug 30;8(8):e1002937. doi: 10.1371/journal.pgen.1002937 (PMC3431340; doi:10.1371/journal.pgen.1002937)
Supplement: Table S1 — Sequences of PCR primers used for mouse genotyping. (DOCX) [file pgen.1002937.s002.docx]

**Table S1.** Sequences of PCR primers used for mouse genotyping

| **Detection of *Spint1* alleles** | | | **Ta=55°C** |
| --- | --- | --- | --- |
| WT Forward | | 5’-ctgtctgtttaccttgtctcg-3’ | |
| WT Reverse | | 5’-taaagggatgttgtcctggg-3’ | |
| KO Forward | | 5’-gagaattcctagaggtaccc-3’ | |
| KO Reverse | | 5’-ggtagttgagggaacatggc-3’ | |
|  |  | | |
| **Detection of *Spint2* alleles** | | | **Ta=55°C** |
| WT Forward | | 5’-aacacatttcaccaccatgc-3’ | |
| WT Reverse | | 5’-ccagactttcctaagtggg-3’ | |
| KO Forward | | 5’-atctgcaacctcaagctagc-3’ | |
| KO Reverse | | 5’-cagaaccagcaaactgaagg-3’ | |
|  |  | | |
| **Detection of *Hgfr* alleles** | | | **Ta=55°C** |
| WT Forward | | 5’-ttaggcaatgaggtgtcccac-3’ | |
| KO Forward | | 5’-cagccgtcagacaattggcac-3’ | |
| WT and KO Reverse | | 5’-ccaggtggcttcaaattctaagg-3’ | |
|  |  | | |
| **Detection of *St14* alleles** | | | **Ta=55°C** |
| WT Forward | | 5’-cagtgctgttcagcttcctctt-3’ | |
| KO Forward | | 5’-gcatgctccagactgccttg-3’ | |
| WT and KO Reverse | | 5’- gtggaggtggagttctcatacg-3’ | |
|  |  | | |
| **Detection of *Prss8* alleles** | | | **Ta=64°C** |
| WT Forward | | 5’-ccaacggccttcactgtactgt-3’ | |
| *fr* Forward | | 5’-tttcccaacggccttcactgtactga-3’ | |
| WT and *fr* Reverse | | 5’-ccctccatgggacaagagagt-3’ | |
|  |  | | |
| **Detection of *F2r* alleles** | | | **Ta=55°C** |
| WT Forward | | 5’-gacgttcaggaaggctga-3’ | |
| KO Forward | | 5’-tggatgtggaatgtgtgcgag-3’ | |
| WT and KO Reverse | | 5’-aaaatgaaagcgtcctgctg-3’ | |
|  |  | | |
| **Detection of *F2rl1* alleles** | | | **Ta=55°C** |
| WT Forward | | 5’-ggtccaacagtaaggctgct-3’ | |
| KO Forward | | 5’-gccagaggccacttgtgtag-3’ | |
| WT and KO Reverse | | 5’-tcaaagactgctggtggttg-3’ | |
|  |  | | |
| **Detection of *Scnn1a* alleles** | | | **Ta=55°C** |
| WT Forward | | 5’-gtcactgtgtgcacccttaa-3’ | |
| KO Forward | | 5’-ctcaatcagaaggaccctgg-3’ | |
| WT and KO Reverse | | 5’-gcacaaagatcttatccacc-3’ | |
